# Diagnosis and Management of Cervicofacial Actinomycosis: Lessons from Two Distinct Clinical Cases

**DOI:** 10.3390/antibiotics9040139

**Published:** 2020-03-25

**Authors:** Anette Stájer, Barrak Ibrahim, Márió Gajdács, Edit Urbán, Zoltán Baráth

**Affiliations:** 1 Department of Prosthodontics, Faculty of Dentistry, University of Szeged, 6720 Szeged, Hungary; barrakibrahim@gmail.com (B.I.); stoma@stoma.u-szeged.hu (Z.B.); 2 Department of Pharmacodynamics and Biopharmacy, Faculty of Pharmacy, University of Szeged, 6720 Szeged, Hungary; gajdacs.mario@pharm.u-szeged.hu; 3 Department of Public Health, Faculty of Medicine, University of Szeged, 6720 Szeged, Hungary; tidenabru@freemail.hu; 4 Institute of Translational Medicine, Faculty of Medicine, University of Pécs, 7624 Pécs, Hungary

**Keywords:** *Actinomyces israelii*, actinomycosis, cervicofacial, dental, oral, susceptibility-testing

## Abstract

Members of the *Actinomyces* genus are non-spore-forming, anaerobic, and aerotolerant Gram-positive bacteria that are abundantly found in the oropharynx. They are the causative agents of actinomycosis, a slowly progressing (indolent) infection with non-specific symptoms in its initial phase, and a clinical course of extensive tissue destruction if left untreated. Actinomycoses are considered to be rare; however, reliable epidemiological data on their prevalence is lacking. Herein, we describe two representative and contrasting cases of cervicofacial actinomycosis, where the affected patients had distinctively different backgrounds and medical histories. Identification of the relevant isolates was carried out using matrix-assisted laser desorption/ionization mass spectrometry; antimicrobial susceptibility was performed using E-tests. Cervicofacial actinomycoses are the most frequent form of the disease; isolation and identification of these microorganisms from relevant clinical samples (with or without histological examination) is the gold standard for diagnosis. The therapy of these infections includes surgical debridement and antibiotic therapy, mainly with a penicillin-derivative or clindamycin.

## 1. Introduction

Species in the *Actinomyces* genus are non-spore-forming, anaerobic, and aerotolerant Gram-positive bacteria, belonging to the *Actinomycetales* order [1]. These bacteria are abundantly present in the normal microbiome of humans, especially in the oropharynx (namely, in periodontal pockets, gingival crevices and tonsillar crypts, while also presenting on carious teeth, in dental plaques and biofilm) [2]. *Actinomyces* spp. are the causative agents of actinomycosis, a slowly progressing (indolent) infection with non-specific symptoms in its initial phase, and a clinical course of extensive tissue destruction, if left untreated [3]. Actinomycoses is considered to be rare (with a reported annual incidence of 1/300,000 persons, based on the reports available in the literature); however, reliable epidemiological data on their prevalence is lacking [4,5]. The diagnosis of this disease is frequently delayed, as the clinical presentation can mimic other pathologies, such as malignancies, active *Mycobacterium tuberculosis* infection, nocardiosis, fungal infections, infarctions (e.g., in the lungs), or other granulomatous diseases [6,7]. In addition, the clinical presentation of individual infections is myriad, further complicating diagnostic processes. Based on the affected anatomical region, these infections may be divided into cervicofacial, abdominal, thoracic, pelvic, and cutaneous actinomycoses [2,8]. Cervicofacial *Actinomyces* infections (including presentations with central nervous system involvement), also called as “lumpy jaw syndrome”, are the most common type, accounting for 40%–60% of cases overall; the upper and lower mandibles (50%), the cheeks (10%–15%), and the chin (10%–15%) are most frequently affected [8,9]. 

Forty-nine different species have been described in the *Actinomyces* genus, out of which 27 species have been implicated as causative agents in human infections: in cervicofacial actinomycosis, *A. israelii*, *A. odontolyticus,* and *A. meyeri* are most frequently implicated (>90% of cases); nevertheless, other species, such as *A. dentalis*, *A. hominis*, *A. oris*, *A. pyogenes,* and *A. viscous,* are also relevant cervicofacial pathogens [8,10]. Cervicofacial actinomycosis was reported more frequently in patients living in rural areas, compared to people living in urban environments (the observed prevalence was 10:1); this difference was usually attributed to poor hygiene, neglected health status, low socioeconomic status and close contact with animals [11]. Histopathological examinations, microbiological culture, and consideration of the patient’s medical history and underlying illnesses, together with imaging methods are all important components for the definitive diagnosis of actinomycoses [12,13,14]. However, in some instances and for many healthcare-settings, histopathology is not available or not routinely performed; thus, a presumptive diagnosis is carried out from the symptomatology and microbiological findings.

## 2. Cases: Local Epidemiology

There is virtually no data available on the prevalence of cervicofacial actinomycosis in Hungary, and because these infections are not listed for surveillance in the National Bacteriological Surveillance in Hungary or the WHO Recommended Surveillance Standards, clinicians may only rely on estimations based on international data. To amend this, according to the aim of this paper (described in the following section), two representative and contrasting cases are described that have occurred in our Faculty of Dentistry, University of Szeged, highlighting some aspects of this pathology in the local context, where histopathology was not performed as a part of the diagnostic process. In addition, the local epidemiology in our institution regarding cervicofacial actinomycoses between 2005 and 2015 is also described. The present paper would like to reinforce that cervicofacial actinomycosis is a relatively rare pathology, which is still a major diagnostic challenge, occurring in patients with distinctively different socio-economic backgrounds and past medical histories. These observations are illustrated by our two presented cases.

### 2.1. Case No. 1

A 22-year old female patient presented at the dental clinic with complaints that she attributed to a previous dental procedure. She had a history of a lower first molar tooth root canal treatment, five days before presenting in our institute. Two days following the root canal treatment, she reported swelling on the left side of her face, trismus, and difficulty swallowing. The patient received non-steroid anti-inflammatory (NSAID) drugs, while antibiotics were not administered for the previous dental procedure. On examination, asymmetric face and palpable pterygomandibular swelling were noted. Extraorally, the submandibular area was stiff, hard to the touch, and sensitive (the patient reported intense pain), the shape of the jaws were intact and the interincisal distance was less than 1 mm. A part of the left pterygomandibular fold cambered and pushed the uvula to the right side. The movement of the tongue was uninhibited and free in every direction. No fever of lymphadenopathy was present, and apart from the complaints, the patient’s status was generally good. Radiographic examination revealed no specific findings. No underlying disease or pharmacotherapy was found in the patient’s history that would indicate immunosuppression, although the patient reported a penicillin allergy. The patient was otherwise of high social class and presented with generally good oral hygiene, except for the lower left quadrant of the mouth, which was near to the site of the previous dental procedure. 

Because the oral pathology was stiff to the touch (an abscess would be soft to the touch and it would react to pressure) and the patient’s general condition was good (an abscess would generally correspond to fever and/or malaise), the diagnosis of an abscess was considered less likely. With a presumptive diagnosis of cervicofacial actinomycosis, the therapeutic strategy consisted of two steps: initially, extraoral incision, drainage, and curettage was performed. During the incision, the presumptive diagnosis was verified phenotypically as characteristic sulfur granules were seen in the pus, confirming our suspected diagnosis. For this reason, histopathology for this patient was not performed. The extraoral stiffness noted among the initial symptoms persisted after the incision and it only slowly resolved. Before the onset of any kind of antimicrobial therapy, samples were sent to the Department of Bacteriology for examination. Two days later, general anesthesia was introduced, and subsequent examination showed pericoronitis and gingival inflammation around the lower left wisdom tooth. This was the area of the mouth that was noted to be neglected regarding dental hygiene during previous examinations. In this area, a large amount of plaque was noted, which may provide ideal conditions for the cultivation of pathogenic bacteria. Necrotic tissue was removed with an intraoral incision from the prerygomandibular space; additionally, the lower-left first and third molars were extracted, and the purulent discharge was drained. 

**Figure 1:**
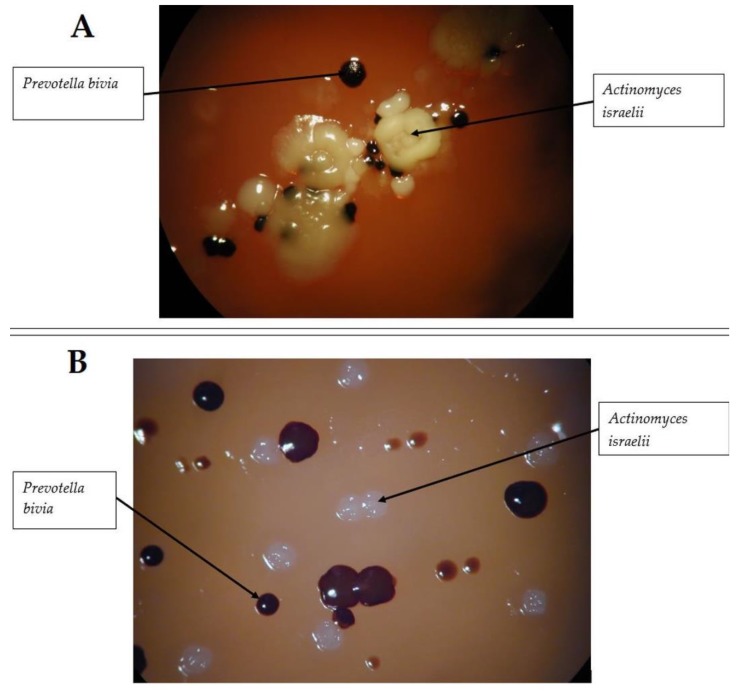
Culture results of the sample taken in Case 1. **A:** Colonies observed on anaerobic blood agar after 5 days of incubation **B:** Colonies observed on anaerobic blood agar after 7 days of incubation (courtesy of Gabriella Terhes PhD, University of Szeged).

The samples sent to the microbiology laboratory were processed and incubated in both aerobic (standard CO_2_ incubator at 37 °C 1–2 days) and anaerobic enviroments (Concept 400 anaerobic incubator, Biotrace International Plc., UK; for 5–7 days in an atmosphere of 90% N_2_, 5% H_2_, and 5% CO_2_ at 37 °C) [15]. In the Gram-staining of the sample, two different morphologies of Gram-positive rods (thich, branching, and a narrower type) and Gram-negative cocco-bacilli were observed; in subsequent staining procedures, no fungal hyphae, yeasts, or acid-fast bacteria were noted. Cultivation of the samples yielded the following microorganisms in high colony-forming unit (>10^5^ CFU/ml): *A. israelii*, *Clostridium ramosum*, *C. clostridioforme*, *Prevotella bivia* (strict anaerobes), *Capnocytophaga* spp., and *Eikenella corrodens* (facultative anaerobes) (Figure 1). Identification was performed using matrix-assisted laser desorption/ionization mass spectrometry (MALDI–TOF MS; Microflex MALDI Biotyper (Bruker Daltonics, Bremen, Germany)). Antimicrobial susceptibility testing (AST) was performed using E-tests on anaerobic blood agar plates, the interpretation of the results was based on EUCAST breakpoints (http://www.eucast.org/clinical_breakpoints/), taking into account the intrinsic resistance of relevant isolates [15,16,17]. The results of the AST are presented in Table 1. The pathogenic role of *Capnocytophaga* spp. and *E. corrodens* was discarded, after consultation with clinical microbiologists. Based on the susceptibility results and the anamnestic data, the patient received clindamycin 300 mg/q8h (every three hours) intravenously for 7 days, supplemented with per os metronidazole 500 mg/q12h (every twelve hours). On the 7th day of therapy, the patient was discharged from the clinic, with instructions to take per os clindamycin 300 mg/q8h for an additional 7 days; in addition, the dentists gave additional instructions to the patient regarding oral hygiene. On the follow-up examination, three days after the finishing of the antibiotic therapy, the patient had no complaints. The patient had no complaints after the 1-month follow up, while she did not show up for the check-up 3 and 6 months later.

### 2.2. Case No. 2

**Figure 2:**
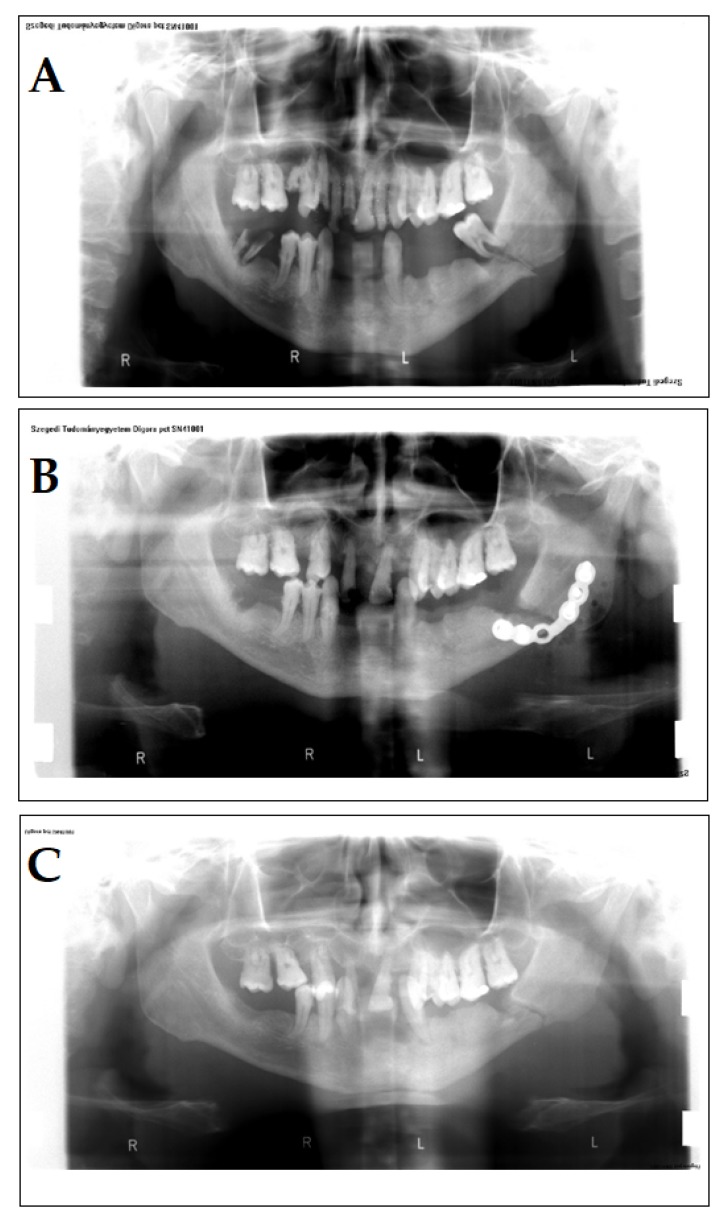
Panoramic X-rays of the patient in Case 2. **A:** Initial status of the patient, lower left molar in the fracture. **B:** Osteosynthesis with Leibinger-plate. **C:** Following the removal of the Leibinger-plate, during the healing period.

A 40-year-old male patient presented in our clinic with complaints that he attributed to a previous accident. In the past medical history, the patient reported that a wooden log hit his face. The chief complaints of the patients were pain and swelling on the left side of the face, paresthesia on the lower lip from the midline to the left side, and a wound with serosanguineous discharge. A few days previously, he reported seeing his general practitioner; there, the wound was rinsed with Betadine (povidone-iodine) several times; however, healing of the wound was not complete. No underlying disease or pharmacotherapy was found in the patient’s history that would indicate immunosuppression, and he did not receive antibiotics either. On examination, a 5-mm fistula was detected on the skin of the face, in addition to partial trismus. A panoramic X-ray was performed, where a mandibular fracture was identified (Figure 2A). The patient was of lower social standing and had neglected oral hygiene and an incomplete set of teeth (Figure 2A–C).

The surgical therapy of the patient was performed under general anesthesia: the surgeons operated on the submandibular part of the bone, extracted the tooth, which was in the broken-line, and removed the bone sequestra and the inflamed tissue in the process. After checking the occlusion, the broken edges were reponated and an osteosynthesis was performed using a Leibinger-plate (Figure 2B). On the 4th day post-op, the patient was discharged and was prescribed amoxicillin 750 mg/q12h for 5 days and was instructed to return for suture removal. Upon consultation, it was suspected that the adherence of the patient towards taking the prescribed antibiotics was inadequate. The patient came back 6 months later, complaining of swelling and pain on the same part of his face. Partial trismus was once again seen, and on the submandibular part of his face (where the surgical callus was found), a fistula was detected. An incision was made in this area and a sample was taken to be sent to the Department of Bacteriology for examination. While the Gram- and other staining methods (acid-fast, lactophenol blue) were inconclusive, cultivation of the sample in an anaerobic environment (for metholodological details, see Case 1) yielded *A. israelii* in high colony counts (>10^5^ CFU/ml) as the only isolated pathogen; other anaerobes, facultative anaerobes of fungi were not detected. As *A. israelii* was the only isolated species and the symptoms of the patient corresponded to the presumptive diagnosis of cervicofacial actinomycosis, histopathology was not performed. The therapy of the patient included 1200 mg/q8h amoxicillin intravenously, and 8 days later, the Leibinger-plate was surgically removed. The surgeons also drained the infected site. The patient was discharged and he was instructed to take amoxicillin 750 mg/q12h for 7 days per os. The patient was continuously observed for a year (monthly for three months and then every three months); however, there were no additional complaints.

### 2.3. Local Epidemiological Snapshot

At the Faculty of Dentistry, University of Szeged (responsible for the specialized dental care in the Southern region of Hungary), the prevalence of verified (with microbiology results or histological findings) cases of actinomycoses is rare; in most cases, the diagnosis of this pathology is presumptive and is aided by the corresponding symptoms and clinical findings of these patients. At our Institution, there were 40 patients between 2005 and 2015, where the diagnosis of actinomycosis was made based on past medical history and clinical presentation of the patients; in 32 out of 40 cases (80%), subsequent microbiological results confirmed our diagnosis, in 10 out of 32 cases, *Actinomyces* spp. was the only isolate from the clinical sample, while in 22 out of 32 cases contained *Actinomyces* spp. in the form of a mixed aerobic–anaerobic flora. In the remaining 8 cases, no bacterial or fungal species were detected. In 29 out of 40 cases (72.5%), an underlying dental pathology/odontogenic problem or a previous dental intervention was noted in the anamnestic data of affected patients. Only two patients were suffering from cancer, the others presented with no underlying immunosuppression. Twenty-six out of 40 (65%) patients were aged between 40 to 50 years at the time of the infection. In most cases, patients were characterized by bad oral hygiene, only 6 out of 40 patients (15%) had teeth in good condition and appropriate oral hygiene. The localization of the actinomycetous lesions were predominantly submandibular–perimandibular (in 50% of cases).

## 3. Discussion

In the present report, we discuss two cases of cervicofacial actinomyces infections from patients with distinctly different backgrounds and medical history. The first patient was a healthy, young female with a generally good oral hygiene, affected by the disease, following a dental procedure. The second case involved an older male, following an injury, which was probably facilitated by the bad oral hygiene of the patient. In addition, the results from our epidemiological survey were in line with the literature findings: most patients had bad oral hygiene and the patients between ages 40–50 years were predominantly affected. Infiltration of *Actinomyces* spp. through damaged mucosal surfaces (caused by medical interventions, trauma, immunosuppression, or cancer) have been described as a principal factor for the development of the disease [8,18]. These infections mainly affect patients between 20 to 60 years of age, with a peak in incidence around 40 to 50 years; developments in hygiene practices and the use of prophylactic antibiotic therapy following dental procedures had a significant role in curbing the prevalence of actinomycosis; however, an increase in their frequency (proportionate with the increase in the number of immunosuppressed patients) in the last two decades is worrisome [2,3,19]. If caught early on and treated appropriately, cervicofacial infections usually resolve without sequelae. However, rare presentations of this disease must also be taken into consideration [20]: in a recent case report from Hungary, *A. turicensis* was a causative agent of meningitis, following a purulent mastoiditis; unfortunately, the patient died from the complications of this infection [21].

In an Italian case report, a patient with a similar presentation to Case 1 was admitted, with no history of maxillofacial trauma or dental procedure; in their case, *Actinomyces* spp. was also isolated as a part of a polymicrobial flora (including *Fusobacterium nucleaum*, *P. asaccharolytica,* and *S. aureus*) and the diagnosis was made based on the presence of the characteristic sulfur-granules in the pus and imaging methods. The patient was treated with high-dose penicillin for 4 weeks and made a complete recovery [22]. Ayoade et al. reported two distinct cases of periapical actinomycosis, which is a very rare presentation of the cervicofacial form [12]: both of these patients had no history of dental procedures but presented with underlying immunosuppression (due to type 2 diabetes and multiple myeloma, respectively) and they were over 60 years of age. In both cases, the diagnosis was based on histopathological examination using hematoxylin and eosin and Gomori–Grocott methenamine silver stains, where both the bacteria and the sulfur granules were shown [12]. In the case series presented by Moghimi et al. [23], 19 cases of cervicofacial actinomycosis cases were characterized: all patients complained of swelling, while 17 also had severe pain. Most patients (14 out of 19) were treated with antibiotics for 8–23 days (predominantly with β-lactam antibiotics), and all cases ended with clinical cure [23]. In a Turkish case report, actinomycosis was verified from an unknown inflammatory lesion in the oral cavity [2], while another report highlighted the potential of these actinomycetous lesions to mimick malignancy in a young (16-year-old), otherwise healthy patient [6]. Another uncommon presentation of this disease in the oral cavity is lingual actinomycosis, which is also a diagnostic challenge, as the patient may not have relevant complaints or restrictions in tongue movements, presenting as a slowly-growing mass [24,25]. 

Isolation and identification of *Actinomyces* spp. from relevant clinical samples (in the presence of the characteristic symptoms) is a critical step in the diagnostic procedure of this disease, but the absence of pathogens from the representative culture samples do not exclude the diagnosis of actinomycosis. [2,8]. The gold standard for the diagnosis of cervicofacial actinomycosis is culture, with or without histological examination of a tissue sample, pus, or abscess [4]. Due to the fastidious nature of these bacteria, prolonged incubation (5–14 days) in an anaerobic environment (which is not available in all routine laboratories) is required. In the meantime, the continuous collaboration and communication of physicians and clinical microbiologists is of utmost importance. However, laboratory confirmation of these infections is often difficult, and the absence of these microorganisms in culture is not useful to exclude their clinical relevance in infections. Previous antibiotic exposure may slow down or eliminate these bacteria if the samples were taken after the onset of antimicrobial therapy [2,8]. In addition, *Actinomyces* spp. are frequently co-isolated with commensals (depending on the site of infection and sample type): *Aggregatibacter actinomycetemcomitans*, *Bacteroides* spp., *Capnocytophaga* spp., *E. corrodens*, *Staphylococcus* spp., *Streptococcus* spp., *Veilonella* spp., and members of the *Enterobacteriaceae* family [26]. In histological examinations, the presence of characteristic sulfur granules may also be indicative of actinomycosis; however, in almost 50% of cases, these granules are absent [27]. A brief summary of the diagnostic hallmarks of cervicofacial actinomycoses is presented in Table 2. 

The therapy of these infections includes surgical debridement and antibiotic therapy: based on literature data, the first-line therapy of actinomycosis is a standard high-dose intravenous penicillin G (12–24 million U/day for adults) or ampicillin therapy for 2–6 weeks, which should be switched to penicillin V or amoxicillin per os for 6–12 months, to prevent a relapsing infection [2,8,18,19,20,28]. Alternatively, clindamycin (owing to its good tissue penetration) is also a viable first-line option for therapy [2,8]. In case of a polymicrobial infection, metronidazole (against other anaerobes), β-lactam/β-lactamase-inhibitor combinations or carbapenems, aminoglycosides (e.g., for *Enterobacteriaceae*) should be considered in the therapeutic regimen. Nevertheless, more recent reports indicate that long-term antibiotic-therapy (especially in mild cases) may not even be necessary, as there was no significant difference observed in the clinical outcomes associated with the therapies of different duration [22,23,29]. 

Resistance levels in anaerobes are generally considered to be predictable, for this reason (in addition to their fastidious growth requirements and financial constraints), antimicrobial susceptibility testing is not routinely performed for *Actinomyces* spp. However, species-level identification may have relevance in the future, as *bla*_TEM_-type β-lactamases were detected in *A. graevenitzii* and *A. europaeus*, with isolates showing resistance to ceftriaxone and piperacillin-tazobactam [30,31]. The advances in routine and rapid microbiological methods in anaerobic bacteriology, such as polymerase-chain reaction, MALDI–TOF MS and sequencing will probably aid the diagnostics of these rarely-occurring, neglected infections [11,32].

## 4. Conclusions

As our cases have demonstrated, the correct clinical diagnosis of this rare disease is sometimes difficult, due to its non-specific symptoms and the fastidious, slow-growing nature of *Actinomyces* spp., requiring an anaerobic atmosphere and advanced level of microbiological laboratory background. Our first case is eye-catching, because the patient had no fever or lymphadenopathy, and apart from the mild complaints, the patient’s status was generally good. Radiographic examination revealed no specific findings, no underlying disease or pharmacotherapy was found in the patient’s anamnestic data that would indicate immunosuppression. This young female patient was otherwise well educated, of high social status, and presented with generally good oral and personal hygiene. In contrast, the case of the second patient also deserves attention, because he had a well-documented, but, for a long time, neglected prior accident. This patient was of lower social standing and had neglected oral hygiene and an incomplete set of teeth. After the first surgical intervention, the patient came back six months later, complaining of sharp pain and swelling on the same part of his face. This very serious case draws attention to the inadequate patient cooperation, the use of antibiotics in the proper dosage, and for a right period of time after a surgical procedure. In light of the diagnostic difficulties, cervicofacial actinomycosis has been referred to as the great masquerader of head and neck disease according to the literature data: fewer than 10% of infections are correctly diagnosed according to the literature data; this observation is also illustrated by our case reports.

## Figures and Tables

**Table 1:** Antimicrobial susceptibility testing results for strict anaerobes in Cases 1 and 2.

Tested Antibiotics	Benzylpenicillin	Amoxicillin	Piperacillin-Tazobactam	Imipenem	Meropenem	Clindamycin	Vancomycin	Metronidazole
***Case 1***	Minimum inhibitory concentrations (MIC; mg/L)							
*A. israelii*	0.125 (S)	1 (S)	1 (S)	0.25 (S)	0.125 (S)	1 (S)	0.064 (S)	R*
*C. ramosum*	**2 (R)**	8 (S)	4 (S)	0.125 (S)	0.125 (S)	1 (S)	0.125 (S)	0.25 (S)
*C. clostridioforme*	**1 (R)**	8 (S)	8 (S)	0.25 (S)	0.125 (S)	1 (S)	0.125 (S)	0.125 (S)
*P. bivia*	**1 (R)**	0.125 (S)	1 (S)	0.25 (S)	0.25 (S)	2 (S)	R*	0.25 (S)
***Case 2***	Minimum inhibitory concentrations (MIC; mg/L)							
*A. israelii*	0.125 (S)	0.5 (S)	0.5 (S)	0.125 (S)	0.125 (S)	1 (S)	0.064 (S)	R*

Interpretative criteria were based on EUCAST standards. S: susceptible; R: resistant; R* = intrinsic resistance; values in boldface represent resistance (based on MIC values) or intrinsic resistance.

**Table 2:** Hallmarks of the diagnosis of cervicofacial actinomycoses (based on [8])**.**

Clinical Suspicion	Culture	Histopathology and Imaging
Identification of relevant risk factors (general and disease-specific)	Taking appropriate samples for anaerobic processing	Presence of sulfur granules
Patient’s medical history	Prolonged incubation (5-14 days) in anaerobic environment	Utilization of staining methods (PAS, hemtoxyllin–eoisn, Gömöri–Grocott’s methenamine silver, fluorescein-conjugated antibodies)
Presence/absence of chronic granulomatous lesions	Gram-staining	Imaging (radiography, ultasound, CT, MRI if relevant)
Consideration of differential diagnoses	Utilization of biochemical (API20/VITEK, ANI card) and next-generation (MALDI–TOF MS, PCR, sequencing) identification methods	
	Differentiation of commensal strains from true pathogens	
